# Psychometric properties of fertility desire scale (FDS) developed for Iranian parents

**DOI:** 10.1186/s12889-019-7413-x

**Published:** 2019-08-14

**Authors:** Seyed Abolhassan Naghibi, Maryam Khazaee-Pool, Mahmood Moosazadeh

**Affiliations:** 1 Department of Public Health, School of Health, Mazandaran University of Medical Sciences, Sari, Iran; 2 Health Sciences Research Center, Addiction Research Institutes, Mazandaran University of Medical Sciences, Sari, Iran

**Keywords:** Fertility desire, Psychometrics, FDS

## Abstract

**Background:**

Fertility choice is a critical women’s right. Although researchers have studied the positive effects of fertility desire, its different components have, unfortunately, been neither established nor implemented for parents. A reliable and valid scale is required to measure this vital aspiration of a couple. This study aims to develop and evaluate the psychometric properties of the Fertility Desire Scale (FDS), which is designed to assess fertility desire among Iranian parents.

**Methods:**

A multi-phase instrument developmental approach was used to develop this scale in 2017. The items for the questionnaire were generated using three approaches: a qualitative study, an interview with an expert panel, and a comprehensive literature review. To provide a draft form of the questionnaire, we performed face and content validity analyses. The questionnaire validation was conducted on a sample of married women and men, recruited from public places affiliated with the Mazandaran province. Finally, measurement and analyses of exploratory and confirmatory factors, internal consistency reliability, item-scale correlation, and test-retest reliability of the questionnaire were performed to complete the validation process.

**Results:**

Thirty-five items were initially developed on the basis of the interviews with the expert panel and the literature review. The questionnaire was subsequently reduced to include 27 items after performing the content and face validity testing. The exploratory factor analysis (EFA) identified four factors (positive childbearing motivations, preferences, childbearing worries, and social beliefs) comprising 19 items that jointly accounted for 55.44% of the observed variance. The confirmatory factor analysis (CFA) also revealed the suitable model fit for the data. The Cronbach’s alpha coefficient for the subscales ranged from .83 to .86, and the intraclass correlation coefficient (ICC) ranged from .88 to .92; these coefficients are well above the acceptable thresholds.

**Conclusion:**

Results from this validation study demonstrated that the FDS is a valid and reliable questionnaire for measuring fertility desire that can be used in clinical practice, as well as in similar future studies.

## Background

Prior research suggests that total fertility rates are decreasing, in developed and developing countries [1, 2], although, in many developing countries, including Iran, the trend reflects either stable or decreased fertility rates [3]. Generally, total fertility rates fell from 4.97 children per woman, in the years between 1950 and 1954, to 2.53, from 2005 through 2010; over this same time period, developing countries experienced even more apparent changes in the total fertility rate, which dropped sharply from 6.08 to 2.69 [1]. The desire to have more children, meaningfully linked to the strong cultural tradition privileging large families [4] and the desire for sons instead of daughters [5], along with a concurrent adoption of a reduced usage of contraception [6], have all contributed to Iran’s relatively higher fertility rates.

Support for this finding include a review of reproductive preferences in Iran, based on a case study showing that, over the past three decades, particularly from 1986 through 1996, there has been an unprecedented decrease in the fertility rate and desired number of children in Iran, and, from 2003 through2006, Iran joined the numerous countries already exhibiting lower fertility rates [7]. In fact, starting in 2006, Iran’s total fertility rate has been decreasing at an alarming rate. By contrast, between 2006 and 2011, the annual population growth rate increased from 2.6 to 29.1. As reported in the Iranian population census of 2011, the total fertility rate peaked at 7.1 children per woman. Subsequently, the overall fertility rate of the Iranian population gradually decreased, to 1.69 children per woman in 2015, and then 1.61 children per woman in 2018 [8].

In recent years, the decline in fertility rates squared with demographic and social factors that must be addressed and resolved. Due to massive changes in the population over the past three decades, fertility plays a crucial role in on the population growth, composition, and demographic structure, and plays a critical role in accelerating population growth. It is therefore essential to develop an objective method to measure fertility [9]. Fertility desire is susceptible to influence by diverse economic, cultural, and social factors that may impact several communities in different ways. As such, socio-demographic topics are a complex and significant issue that often resist explanation by a single model or plan.

Consequently, the impact of the abovementioned factors on different populations can produce a variety of outcomes at different times and in diverse contexts. Reflecting on the social aspect leads to the conclusion that fertility desire has been driven by social-cultural forces and the necessity to sustain the union of a given couple [4]. There is no doubt that the birth of a child is a significant event in the life of any parent, which in turn affects many aspects of life, such as health, economic status, well-being, and family culture [7–9].

Research, in a variety of forms, has revealed that the desire for fertility is subject to a few critical factors, including the following: higher employment rates among woman; women’s economic and social independence; other economic factors, the lack of provisions by government welfare institutions; certain inappropriate attitudes, higher education levels; women’s greater participation in economic, social, and cultural issues of society; disparities in social and cultural characteristics of women in these areas; the higher percentage of women who are 35 years of age and older at the time of matrimony; the number of children borne of each women; the ages of migrant couples; the typical age of women at the first time of childbearing; and women’s self-esteem (physical, social, and psychological) [7, 9–14]. In a 2014 study by Enayat, on the relationship between cultural globalization and desire for childbearing, from among all the elements of cultural globalization, the usage of new information and communication tools, increased awareness and use of approaches to family, the use of mobile phones and associated devices, and individualism, were all determined to have a significant and negative association with the desire to bear children [15].

However, the implementation of any fertility program requires consideration of the desire of partners and the elimination of barriers to behaviors to raise fertility desire [7, 9]. Given the significant role of knowledge and attitudes toward the enhancement of fertility desires, it is essential to apply valid and reliable scales for their evaluation. Although the assessment of fertility desire plays a significant role in measuring the overall quality of health care service delivery systems, only a few studies were ever conducted to evaluate fertility desire [16]. Additionally, the extant scale for this group of subjects consists of an anonymous self-administrated scale that has never been sufficiently validated [13–15]. Consequently, the development of effective interventions for studying and heightening fertility desire requires the development of instruments capable of determining fertility desire among parents. We therefore designed this project to develop a valid and reliable tool, the fertility desire scale (FDS), which, hopefully, can identify suitable contexts for the implementation of interventions aimed at increasing fertility rates among parents and, at the same time, improving their general well-being.

## Methods

### Study design

The project was implemented in two separate phases. Initially, in the first phase, generation of the items and development of the questionnaire development were undertaken, on the basis of the interview with panelists and respondents, as well as a review of the literature. Then, face and content validity testing was performed. In the second phase, the psychometric properties of the developed tool were assessed via a cross-sectional study on a sample of married women and men. To complete this latter phase, we conducted the exploratory factor analysis (EFA), confirmatory factor analysis (CFA), and analyzed item-scale correlation. At the conclusion of the second phase, the reliability of FDS was assessed, using internal consistency (via Cronbach’s’ alpha coefficient) and stability of FDS, via test-retest. Table 1 provides descriptive characteristics of the sample over the two stages.

**Table 1:** Characteristics of the study sample

		EFA sample (*n* = 270)	CFA sample (*n* = 190)
		Number (%)	Number (%)
Age (years)			
	< 20	2 (.7)	8 (4.21)
	20–29	117 (43.33)	111 (58.42)
	> 29	151 (55.90)	71 (37.37)
	Mean (SD)	30.39 (5.23)	27.72 (5.31)
	Range	19–59	17–55
Age of Marriage			
	< 20	98 (36.30)	58 (30.53)
	20–29	164 (60.74)	122 (64.21)
	> 29	8 (2.96)	10 (5.26)
	Mean (SD)	21.53 (3.89)	21.95 (4.09)
Sex			
	Male	24 (8.9)	39 (20.5)
	Female	246 (91.1)	151 (79.5)
Employment status			
	Housewife	164 (60.74)	114 (60)
	Employed	106 (39.26)	76 (40)

### Phase 1: item generation and scale development

#### Research design

This part of the study aimed to develop an instrument to assess fertility desire among Iranian married women and men. We derived items using the following three approaches: identification of the appropriate number content domain, generation of items, and construction of the scale [17]. The content domain refers to content linked to the variables being calculated [18]. In this project, the content domain development derived from a review of the relevant literature in the area of interest, as well as on interviews with the experts and respondents. The data gathered from the interviews with respondents were used to develop the scale items [19, 20]. The literature review represents another approach used to specify the content domain [19]. In the second step toward scale development, item generation was undertaken, in accordance with the feedback gleaned from the content domain. The next phase of the project commenced the process of scale construction, in which items were refined and appropriately organized. All confirmed items were thereby prepared in an operational form [21].

In conclusion, all data obtained from the interviews and literature review were cross-checked, including the 35 items initially generated for inclusion in the scale. Each item was rated on a 5-point Likert scale, ranging from the 1 (*completely agree*) to 5 (*completely disagree*). Next, face and content validity were established.

### Validity

#### Face validity

The scales face validity determines the extent to which a scale seems valid, based on the topics proposed by future respondents. Thus, the establishment of face validity should be prioritized as the first step in the scale validation process [22]. In this study, both qualitative and quantitative face validity were implemented. A sample of married people (*n* = 10) were asked to assess each scale item, and to report whether they experienced any difficulties in responding to the questions posed in the scale. Pursuant to participants’ feedback, some changes were made to the wording of questionnaire items as follows: in item 2, “Someone who has a child receives God’s mercy” was changed to “Having a child at home is a source of God’s blessing”; in item 3, “Having a child gives you support to help you in old age” Was changed to “Having a child is necessary for the maintenance of parents in old age”; and in item 11, “Having children will give you a good chance of making life easier ” was changed to “Having more children will make life happier.”

To measure the quantitative face validity, the item impact score (impact score = frequency × importance) was assessed to display the percentage of married people who regard each item as important or quite important on a 5-point Likert scale. Items with an impact score of 1.5 or greater were deemed satisfactory (which signifies correspondence with a mean frequency of 50% and a mean importance of 3 on the 5-point Likert scale). Items that met such quantitative criteria were retained for inclusion in the questionnaire; other items were omitted [23]. Generally, two items had impact scores lower than 1.5, and 33 items had impact scores ranging from 1.6 to 5. As such, the first version of the tool consisted of 33 items.

#### Content validity

After measuring the questionnaire’s face validity, qualitative and quantitative content validity were evaluated for the remaining 33-items of the newly developed scale. To establish qualitative content validity, a panel of 10 specialists, including a health educator, and experts in obstetrics and gynecology evaluated the initial scale in terms of wording, grammar, and the respective subscales corresponding to each item. To establish quantitative content validity, the content validity index (CVI) and content validity ratio (CVR) were assessed. The specialists were asked to evaluate each item in terms of its accuracy, simplicity, and clarity, on the basis of the CVI [22, 23]. To quantify the CVI, the same specialists were asked to rate each item on a 4-point ordinal Likert scale. Possible responses ranged from 1 (not relevant, not clear, and not simple) to 4 (very relevant, very clear, and very simple). The CVI was calculated as the number of items that achieved an estimated value of 3 or 4, as scored by the specialists [24]. *A* value of .80 or above was deemed satisfactory for CVI [25]. The items’ essentiality was then assessed via the CVR. Each item was rated a 1 (essential), a 2 (useful but not essential), or a 3 (not essential) [24]. The CVR of each item was calculated using the following formula: CVR = [N_e_ – (N/2)] / (N/2). In this formula, N_e_ signifies the number of panelists who score the intended item as “essential” and N is the total number of specialists in the expert panel. To determine the cut-off point and numeric value of CVR, Lawshe’s table was used. For instance, in the present study, which relied on ten expert panelists, if the CVR exceeded 0.62, then that item was recognized as having an acceptable level of significance for inclusion in the scale [26].

An acceptable level of agreement was established (a CVI equal to 0.8 or greater) among specialists, which suggests that the instrument has respectable content validity. In total, this phase of the process led to the removal of six additional items, resulting in a 27-item pool for inclusion in the questionnaire. Moreover, the specialists reviewed the scale in terms of linguistic clarity, selected wording, and item attribution. The 27-item pool used in the analyses below consisted of positively worded statements with five response options: 1 (completely agree), 2 (agree), 3 (no comments), 4 (disagree), and 5 (completely disagree). This first draft of the instrument, comprised of 27 items developed via the foregoing procedure, was then subjected to the psychometric analysis in the next phase.

### Pre-finalized draft

The result yielded from performing the above steps, the pre-finalized 27-item version of the FDS, was prepared for the next phases (validity and reliability testing of the instrument).

### Phase 2: psychometric phase

#### The main study and data collection

To assess the psychometric properties of the FDS in a broader context, a cross-sectional study was performed in 2017, in Mazandaran, Iran. A multistage sampling method was used to collect data. First, Mazandaran (the northern region of Iran) was divided into three areas: west, east, and center. Then, subsequent to generation of a list of cities in each area, three cities in the chosen areas were randomly selected. In the next stage of sampling, available sampling methods were used to select participants in selected areas. Sampling was undertaken in public places and crowded areas (parks, shopping centers, etc.) of selected cities, using the convenience sampling method. To this end, eligible members of the sample who visited these places were requested to participate in the study if: one, they desired to contribute to this study; and two, they fulfilled the inclusion criteria. Participants were recruited into the study if any of the following applied: they had been married for more than one year and had no children; they were women who had experienced an interval of more than three years since their last pregnancy; or they were women approaching the end of their good reproductive years (which last from 18 to 35 years of age). Further, women planning for pregnancy (based on target groups for childbirth, according to the Iranian Ministry of Health), literate, and able to give informed consent to participate in the study were also recruited into the study. The sample size was measured according to Gable and Wolf’s reference, which recommends that a sample of five to 10 persons per item is necessary to support a theoretically fixed factor structure for EFA [27]. The minimum required sample size, i.e., 270 (27 items × 10 persons) married people were thereby recruited using these methods. The trained interviewers requested the participants of this study to fill up the newly developed scale after explaining the purpose of the study.

#### Statistical analysis

Several statistical approaches were used to assess the psychometric properties of the FDS. These were deployed in the following order.

### Construct validity

The construct validity was established using exploratory factor analysis (EFA), confirmatory factor analysis (CFA), and item-scale correlation for the 27 items that remained after analysis of the items.

#### Exploratory factor analysis (EFA)

After determining the content validity, the construct validity of 27 items was measured via EFA to identify the main factors of the FDS. Principal component analysis (PCA), with varimax rotation, was used to extract the main factors of the FDS. Additionally, to measure the sampling suitability of the factor analysis, the Kaiser-Meyer-Olkin (KMO) and Bartlett’s test of sphericity were applied. Moreover, the eigenvalues and scree test (i.e., scree plot) were applied, to determine how many factors to retain. One criterion applicable to determining the number of factors to retain is the Kaiser’s criterion, which is based on a rule of thumb. According to Kaiser’s criterion, the criteria used to extract the main factors had an eigenvalue of 1 or more, which classified them as adequately sufficient [28]. There are no hard and fast rules for determining the cut-off point, and the specific approaches applied were influenced principally by our aims for this study. Most literature advises a cut-off point of 0.4, though there are no real methods recommended to make such a determination, and much depends on the scale being applied. By contrast, Comrey and Lee (1992) advised the use of more stringent cut-off points, including 0.32 (poor), 0.4–0.54 (fair), 0.55–0.62 (good), 0.63–0.70 (very good), or 0.71 and more (excellent) [29]. In the present study, scores of.40 and greater were regarded as acceptable for factor loadings.

#### Confirmatory factor analysis (CFA)

CFA analysis was performed to estimate the coherence between the data and structure of the FDS. The model fit was measured using multiple fit indices. As recommended, numerous fit indices were measured, including relative chi-square, goodness of fit Index (GFI), normed fit index (NFI), comparative fit index (CFI), root mean square error of approximation (RMSEA), and standardized root mean square residual (SRMR) were measured [30]. Relative chi-square is calculated as the ratio of chi-square to degrees-of-freedom, and, if the value obtained is below three, it generally satisfies the model fit [31]. The GFI, CFI, and NF scores ranged from zero to 1. However, values equal to or greater than .90 are also generally accepted as satisfying the model fits [32]. An RMSEA value between.08 and .10 signifies a mediocre fit and a value that falls below.08 reflects a good fit. However, a cut-off value close to .06 and a more stringent upper limit of 0.07 appear to be the common values, agreed upon by the specialists in this study [33]. Values for the SRMR range between zero and 1, with well-fitting models reflected by values below .05, although values equal to or greater than 0.08 are also generally predicted as satisfactory [34].

### Reliability

*a. Internal consistency:* The Cronbach’s’ alpha coefficient was used to measure the internal consistency of the whole instrument and each factor of the FDS. A satisfactory value of Cronbach α, demonstrating satisfactory internal consistency of the scale, was deemed regular if it exceeded 0.70. The rest of the relevant classifications are as follows: excellent (> 0.9), good (0.8–0.9), acceptable (0.7–0.8), questionable (0.6–0.7), and of poor internal consistency (0.5–0.6) [35].

*b. Test-retest:* Test-rest reliability was assessed using correlations between the scores at the first point in time and those at the second point of time. It is calculated by having the same respondents complete a survey at two different points in time, to determine the stability of the responses. Test-retest reliability was tested using the intraclass correlation coefficient (ICC). The values ranged from zero to 1, with a value exceeding 0.90 in ICC regarded as evidence of excellent reliability. However, the single-measure ICC was used and scored according to the following classifications: very good reliability (0.75–0.90), good (0.60–0.74), moderate (0.40–0.59), poor (< 0.40), and no reliability (0) [36]. In the present study, a sample of married people (*n* = 30) completed the FDS twice, before and after a 2-week interval, to assess the ICC, and ICC values greater than 0.6 were regarded as acceptable. SPSS version 24.0 was used for statistical analyses [37].

### Scoring

The final version of the FDS is shown in the appendix. Moreover, detailed scoring instructions are also available. Each item is rated on a scale of 1 to 5 to calculate the score for each row. Item numbers 1, 2, 3, 4, 7, 11, 12, 14, 15, and 18 were positively worded, and item numbers 5, 6, 8, 9, 10, 13, 16, 17, and 19 were negatively worded, and appropriate instructions for scoring, as well as reverse scoring, as needed, for- these items are also included in the attachment/Appendix.

## Results

### Construct validity

#### Exploratory factor analysis

**Fig. 1:**
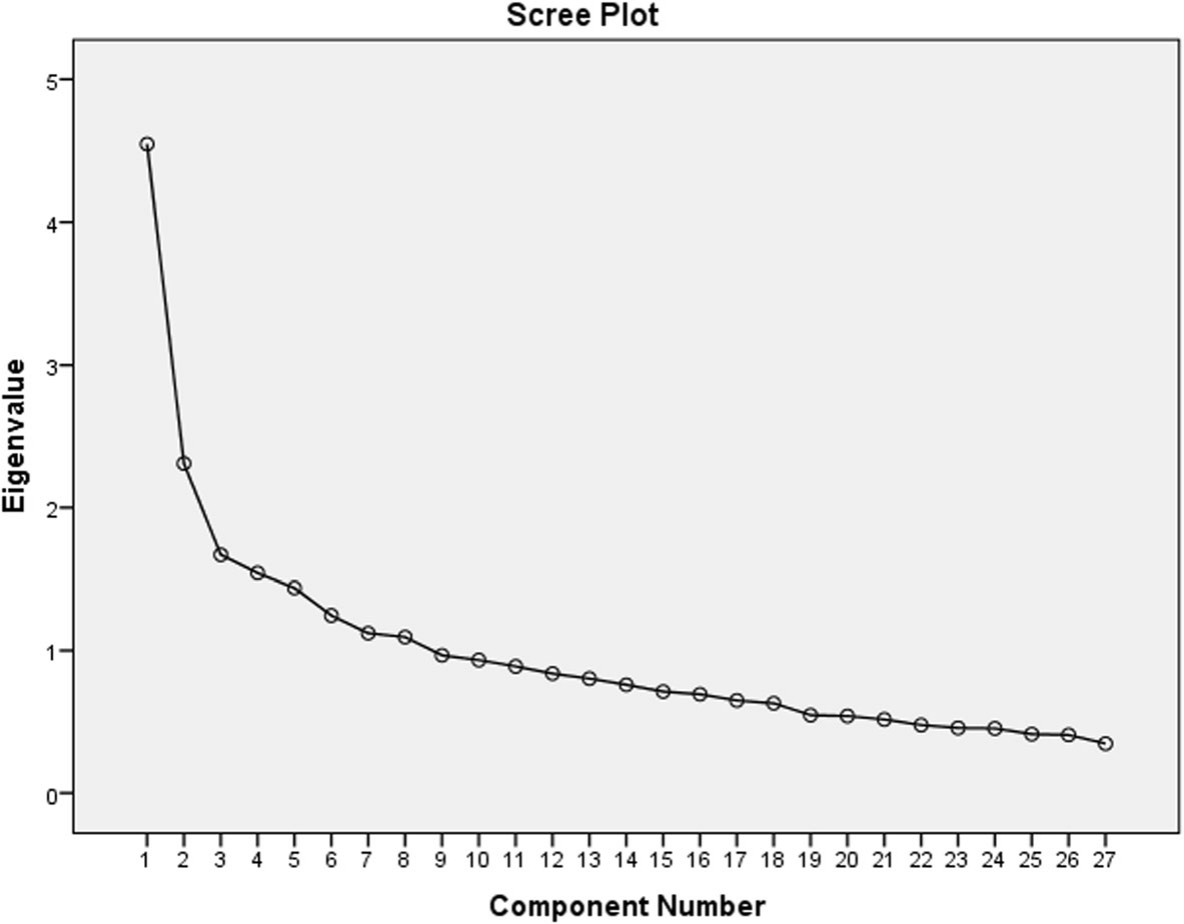
Scree plot for determining factors of the designed instrument

The assessed KMO was .768, and Bartlett’s test of sphericity was significant (χ2 = 1411.591, *p* < .001), showing the suitability of the sample for EFA. A four-factor solution, with a 19-item scale, emerged, based on eigenvalues greater than 1 and a loading level equal to 0.4 or greater. The four-factor solution jointly explained 55.44% of the total observed variance (Table 2). However, the scree plot indicated a six-factor solution (Fig. 1). This factor solution was explored by sequential measurement item performance, conducted by removing the items in a step-by-step process. After removing the items with factor loadings lower than .40, we arrived at a final factor solution that consisted of a 19-item scale, loading on four distinct factors.

**Table 2:** Exploratory factor analysis of the FDS (*n* = 270)

Item	Factor 1	Factor 2	Factor 3	Factor 4
Q2. Having a child at home is a source of God’s blessing.	**.703**	.048	.060	−.117
Q12. Having more children is a source of encouragement in life.	**.632**	−.046	.043	.246
Q11. Having more children will make life happier.	**.628**	−.003	.080	.195
Q7. Preventing pregnancy is an intervention in God’s work.	**.626**	−.036	.195	.041
Q1. Life is meaningless without children.	**.623**	−.093	.038	.016
Q3. Having a child is necessary for the maintenance of parents in old age.	**.623**	−.251	.174	.109
Q4. Having a child improves marital relations between spouses.	**.572**	.115	.280	.005
Q14. In the absence of increased rates of childbirth, the country will experience an increase of the elderly population and a reduction in the workforce.	−.010	**.760**	−.081	−.059
Q15. Single children with no siblings have more psychological problems than children with siblings.	−.169	**.631**	.007	.008
Q18. Our grandchildren should not be deprived of their aunts and uncles.	.022	**.475**	.180	.131
Q8. I think that, if the number of my children was increased, I will not be able to afford to pay their living expenses.	.101	−.097	**.664**	.123
Q5. I think it is out of my power to educate my children properly.	.377	.059	**.642**	.017
Q6. Because I’m already worried about my children’s future, I do not want to have more children.	.033	.459	**.526**	−.084
Q9. I think it’s a heavy responsibility for having children, and I cannot bear it.	−.171	−.030	**−.489**	−.264
Q16. The community more easily accepts smaller families.	−.175	.018	.135	**.713**
Q10. Having a child is not compatible with continuing education.	.400	−.062	−.022	**.537**
Q17. In my opinion, having a stable and secure job is obligatory for childbearing.	.146	.457	−.315	**.516**
Q13. I believe that having fewer children is associated with greater convenience.	.153	.085	.293	**.515**
Q19. I believe that it is necessary to support parenthood ensure that couples have children.	.347	−.362	.244	**.414**

Note. Figures in bold are related to factor loadings equal to or greater than 0.40

As displayed in Table 2, four factors were identified: factor 1 (positive childbearing motivations), which includes 7 items (items 1, 2, 3, 4, 7, 11, and 12); factor 2 (preferences), which includes 3 items (item 14, 15, and 18); factor 3 (childbearing worries), which includes 4 items (items 5, 6, 8, and 9), and factor 4 (social beliefs), which includes 5 items (items 10, 13, 16, 17, and 19). Please refer to the Appendix to read the items of the FDS in full.

#### Confirmatory factor analysis

**Fig. 2:**
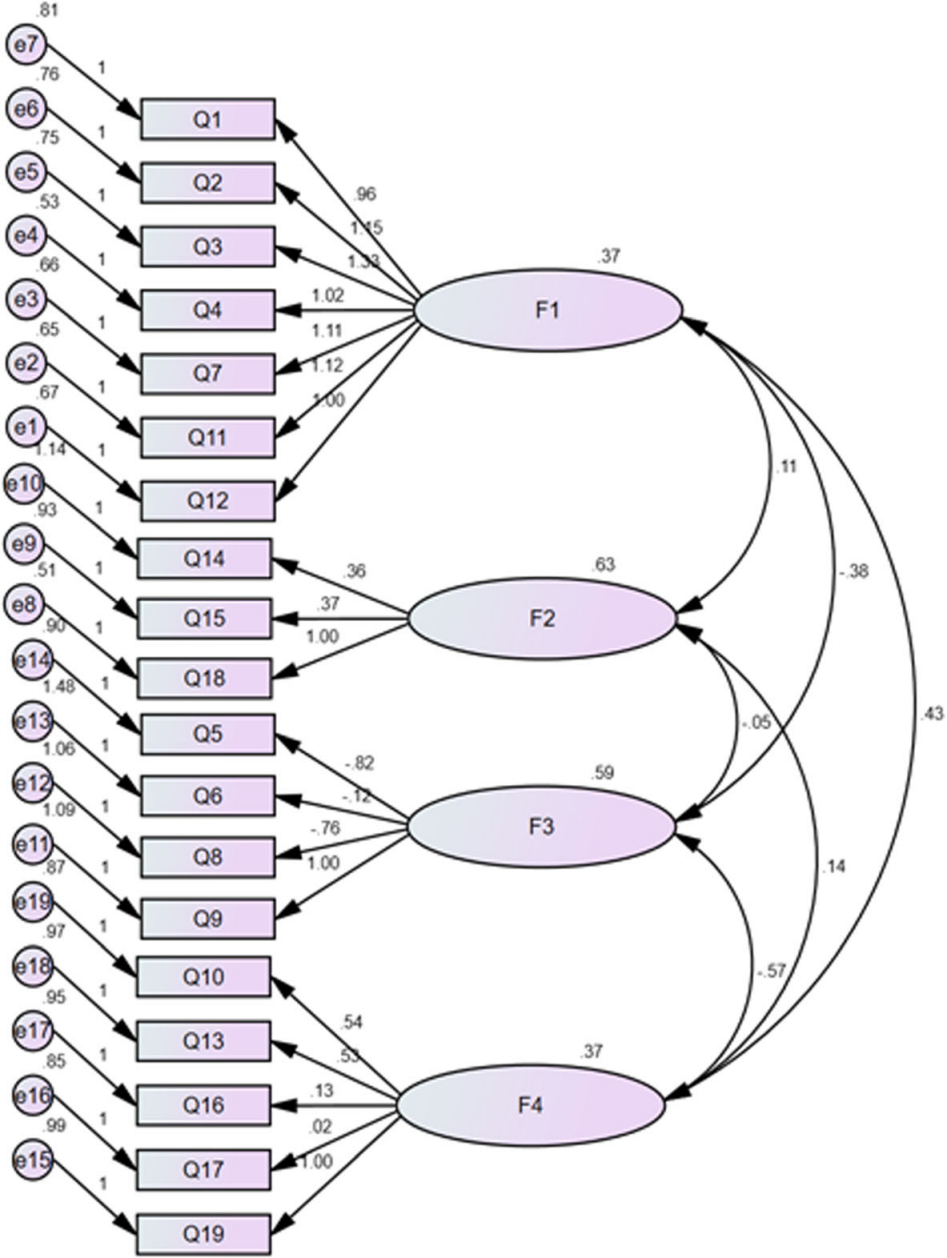
A four-factor model for the scale gained from the confirmatory factor analysis (n = 190)

A confirmatory factor analysis on the 19-item scale was performed to measure the fitness of the model achieved through the EFA. We assessed the covariance matrices and fit indices. Figure 2 demonstrates the good fit of the model. All fit indices were shown to be good. The relative chi-square (χ2/df) was equal to 2.440 (*p* < .001). The RMSEA of the model was .08 (90% CI = .07–.09), and the SRMR was .050. All comparative indices of the model, including GFI, AGFI, CFI, and NFI, were greater than .60 (.83, .79, .74, and .64, respectively), demonstrating a good fit to the data.

#### Reliability

The reliability was measured by the Cronbach’s alpha, for FDS as a whole, as well as for each individual factor of the FDS. The Cronbach’s alpha coefficient for the FDS was .84 and ranged from .83 to .86 for its sub-scales, all of which reflect excellent and high internal reliability. As such, no further items of the scale were eliminated at this stage. Further, the test-retest analysis was conducted to evaluate the stability of the FDS. The results presented acceptable values. Intraclass correlation (ICC) was .89 for the FDS and ranged from .83 to .92 for the subscales of the FDS, thereby substantiating the stability of the scale. The results are shown in Table 3.

**Table 3:** Measures of internal consistency and stability

Factor	The name of factor	Number of items	Cronbach alpha (*n* = 270)	ICC (*n* = 30)
1	Positive childbearing motivations	7 items (1–4, 7,11,12)	0.83	.88
2	Preferences	3 items (14, 15, 18)	0.86	.91
3	Childbearing worries	4 items (5, 6, 8, 9)	0.84	.92
4	Social beliefs	5 items (10, 13, 16, 17, 19)	0.85	.87
Total		19 items	0.84	.89

## Discussion

In this study, we developed a scale, the Fertility Desire Scale (FDS), to measure fertility desire among Iranian married men and women. This is the first project intended to generate a scale aimed at calculating the items linked to fertility desire in Iranian adults. The content of the scale items was initially developed on the basis of an interview with an expert panel and a comprehensive literature review to ensure that this scale covered all theoretical concepts related to fertility desire. In the EFA phase, eight items, with factor loadings below .40, were removed; a four-factor scale of fertility desire emerged, with seven items representing factor 1, three items representing factor 2, three items representing factor 3, and five items representing factor 4. A CFA revealed that the fit of the data was suitable. Reliability testing demonstrated good internal consistency (α > .80). Moreover, we believe that the FDS functions effectively as a new measure to understand the desire for fertility.

Items included in the subscale measuring positive childbearing motivations reflect motivational elements that may encourage couples to make decisions in accordance with childbearing behavior. Motivational components are states that can be stimulated by the environment. Based on Miller’s conception, motivational factors, in this context, stimulate individuals’ fertility-related behaviors that are revealed in continuous processes that generate the mental motivational states of wanting to have children or not. This may give rise to an awareness of the desire to have children or not to have children, which results in the conscious intention to have children or not to have children, which, ultimately, leads to the performance of behaviors instrumental in the avoidance or attainment of childbearing. Consequently, motivational elements include two negative and positive motivational elements [38]. The positive childbearing motivations relate to reasons for wanting to have a child, and negative childbearing motivations link to reasons for not wanting a child [39, 40].

Items included in the preferences subscale aim to elicit the couples’ feelings, perspectives, and desires for reproduction and childbearing. Fertility preferences are complex attitudes, informed by cultural and behavioral contexts, which differ in accordance with a community’s socio-economic development of a community. Additionally, fertility preferences are affected by normative forces, especially in patriarchal societies. Fertility preferences are assessed via statements related to the desire to have more children and the desired family size. The desire (or lack thereof) to have additional children is used to determine the extent to which married women seek to restrict their childbearing [41].

Items included in the childbearing worries subscale refer to the relative significance of negative consequences of having a child, which may discourage couples from making decisions aimed at childbearing. All items in this domain refer to a potential feeling of low self-efficacy regarding an individual’s ability to have more children and/or be an effective parent. Additionally, this subscale includes items that cover a range of reasons for lacking the desire to bear children, which are usually linked to activities that promote decreased fertility potential. Attention to couples’ beliefs and feelings about childbearing is the main focus of maternity health policy. The term “informed choice” reflects that, in addition to the physiological aspects of childbearing, there are also psychological qualities unique to the individual life experiences [42].

According to Bandura’s theory of self-efficacy, an individual factor associated with childbearing is self-efficacy. Individual factors include attitudes and motives related to childbearing, self-regulation, and self-efficacy. Self-efficacy reflects personal beliefs about behavior that influence outcomes. Self-efficacy refers a person’s confidence in himself/herself to perform a particular activity. Individuals’ past experiences affect self-efficacy in terms of understanding the condition at hand, as do vicarious experiences of others, and the degree of emotional and physiological motivation to pursue that activity [43]. A study by Schwartz (2015) showed that high self-efficacy in women promotes a more positive attitude toward motherhood, improving their general health, reducing unnecessary stress during labor, and improving postpartum mental health. This study also noted that mothers with low self-efficacy are likelier likely to change decisions about pregnancy on the basis of complications experienced during previous pregnancies [44].

Items included in the social beliefs subscale involve the social aspects of childbearing from the couples’ perspective. Based on a study conducted by Piltan (2015), who is from Iran, the reproductive act is affected by economic, social, and cultural factors, in addition to the individual elements explained in the previous subscale [45]. Moreover, fertility behavior, which is a social behavior that occurs in a social setting, is affected by environmental elements, as well as the explicit decisions of couples. [46]. Relevant environmental factors include the surrounding pressure and socio-cultural norms in the community [47] related to ensuring the survival of ordinary life before childhood, high participation of women in social activities, as well as social support and an understanding of empowerment process [48]. In total, social beliefs consist of a particular person’s beliefs about what the opinions of society must be. Social beliefs play a substantial role in an individual’s total belief system, due to the direct and indirect impact of the community and the surrounding social environment, such as government, society, religion, organizations and other important people, such as the family and other members of the community. Socio-cultural subjects and disparate expectations from community members may engender critical obstacles to a given individual’s desire to bear children.

Overall, our findings revealed satisfactory psychometric properties for the FDS. The CVI and CVR proved that its content validity was acceptable. Further, the results support the construct validity of its four subscales (positive childbearing motivations, preferences, childbearing worries, and social beliefs), as the results of the EFA and CFA revealed an excellent construction for the FDS instrument. The EFA demonstrated that the four-factor structure of the scale accounted for 55.44% of the total observed variance. The attention to detail employed in the selection of each item of the FDS may be the reason that we have achieved such satisfactory findings.

The internal consistency of the final version of the FDS, as evaluated by Cronbach’s alpha coefficient, was found to be .84, which revealed satisfactory reliability. Additionally, the ICC score showed suitable stability for the FDS, as it was measured by 30 people (men and women) within a 2-week interval (.89). Moreover, we believe that this newly generated instrument may be particularly valuable for health care teams who seek to know and implement procedures that are useful and tailored to specific conditions. The inclusion of four domains in the FDS further allows specialists to recognize specific areas in which a person has needs that should be targeted.

### Limitations

Although the findings of the current study reflect acknowledged several advantages, some limitations must be addressed, as with any research. First, regarding the samples, we only invited people from certain cities in Mazandaran, in the north of Iran; this regional exclusivity probably limited the external validity of the present instrument. The outlooks of our sample cannot necessarily be generalized to the opinions of persons (men and women) from other provinces of Iran. Moreover, although female samples participated in this study in greater numbers than men which may be considered as a limitation, this study showed that the FDS is a valid tool to assess the fertility desires among those with no issues related to infertility.

Consequently, it may be an interesting subject for future study to assess the reliability and validity of the FDS in a sample of men and women, drawn equally from different provinces, cities, backgrounds, or areas. Second, we used two separate samples for our EFA and CFA. Although we used a similar method to collect data from the samples, certain background information of the persons was dissimilar; in particular, the experience of having children, the interval between pregnancies, and the proximity to the end of good reproductive years. This may have affected the findings of the current study, in terms of disparate desires for fertility in these groups. Third, but not least, generalizability to non-Persian attending people cannot be expected; further research, using other ethnic populations, must apply before the tool can be confirmed to support its objective of measuring fertility desire. Repeating the factor structure across various kinds of participants may shed light on the generalizability of the FDS. Similarly, validating the scale with other samples from different provinces may support its usefulness beyond a specific area. Finally, in future studies, it may be interesting to incorporate additional scales that measure people’s desire for fertility to assess the criterion validity of the Persian version of the FDS.

In summary, a central objective of the National Institute of Child Health and Human Development (NICHD) mission for the present century is to address issues related to fertility. To that end, we developed the FDS, which was shown to have satisfactory psychometric properties. The FDS measures factors that affect the desire for fertility that can help to promote a persons’ health because it allow for less pressure from the community to bear children.

## Conclusion

Given the lack instruments to measure fertility desire among couples, the findings suggest that the FDS is a valid and reliable scale. More studies, with participants from other contexts and settings, are needed to strengthen the psychometric properties of the FDS.

## Data Availability

The data sets generated and analyzed during this study are not publicly presented, because of a desire to protect the participants’ anonymity; they are, however, available from the corresponding author on reasonable request.

## Appendix

*Positive childbearing motivations* Q2. Having a child at home is a source of God’s blessing. Q12. Having more children is a source of encouragement in life. Q11. Having more children will make life happier. Q7. Preventing pregnancy is an intervention in God's work. Q1. Life is meaningless without children. Q3. Having a child is necessary for the maintenance of parents in old age. Q4. Having a child improves marital relations between spouses. Preferences Q14. In the absence of increased rates of childbirth, the country will experience an increase of the elderly population and a reduction in the workforce. Q15. Single children with no siblings have more psychological problems than children with siblings. Q18. Our grandchildren should not be deprived of their aunts and uncles. Childbearing worries Q8. I think that, if the number of my children is increased, I will not be able to afford to pay their living expenses. *(reverse-scored)* Q5. I believe it is out of my power to educate my children properly. *(reverse-scored)* Q6. Because I'm already worried about my children’s future, I do not want to have more children. *(reverse-scored)* Q9. I think having children is a heavy responsibility, and I cannot bear it. *(reverse-scored)* Social beliefs Q16. Smaller families are more readily accepted by the community. *(reverse-scored)* Q10. Having a child is not compatible with continuing education. *(reverse-scored)* Q17. In my opinion, having a stable and secure job is obligatory for childbearing. *(reverse-scored)* Q13. I believe that having fewer children is associated with greater convenience. *(reverse-scored)* Q19. I believe that it is necessary to support parenthood ensure that couples have children. *(reverse-scored).*

## Abbreviations

CFA: Confirmatory factor analysis
CFI: Comparative fit index
CVI: Content validity index
CVR: Content validity ratio
EFA: Exploratory factor analysis
FDS: Fertility Desire Scale
GFI: Goodness of fit index
ICC: Intraclass correlation coefficient
NFI: Normed fit index
NICHD: national institute of child health and human development
NNFI: Non-normed fit index
RMSEA: Root mean square error of approximation
SRMR: Standardized root mean square residual
